# Social demonstration of colour preference improves the learning of associated demonstrated actions

**DOI:** 10.1007/s10071-024-01865-7

**Published:** 2024-04-09

**Authors:** Noam Zurek, Na’ama Aljadeff, Donya Khoury, Lucy M. Aplin, Arnon Lotem

**Affiliations:** 1 School of Zoology, Faculty of Life Sciences, Tel Aviv University, Tel Aviv, Israel; 2 Department of Evolutionary Biology and Environmental Science, University of Zurich, Zurich, Switzerland; 3 Division of Ecology and Evolution, Research School of Biology, The Australian National University, Canberra, Australia

**Keywords:** Social learning, Social learning mechanisms, Cognitive mechanisms, Cognitive evolution, Mechanistic constraints, House sparrows

## Abstract

We studied how different types of social demonstration improve house sparrows' (*Passer domesticus*) success in solving a foraging task that requires both operant learning (opening covers) and discrimination learning (preferring covers of the rewarding colour). We provided learners with either paired demonstration (of both cover opening and colour preference), action-only demonstration (of opening white covers only), or no demonstration (a companion bird eating without covers). We found that sparrows failed to learn the two tasks with no demonstration, and learned them best with a paired demonstration. Interestingly, the action of cover opening was learned faster with paired rather than action-only demonstration despite being equally demonstrated in both. We also found that only with paired demonstration, the speed of operant (action) learning was related to the demonstrator’s level of activity. Colour preference (i.e. discrimination learning) was eventually acquired by all sparrows that learned to open covers, even without social demonstration of colour preference. Thus, adding a demonstration of colour preference was actually more important for operant learning, possibly as a result of increasing the similarity between the demonstrated and the learned tasks, thereby increasing the learner’s attention to the actions of the demonstrator. Giving more attention to individuals in similar settings may be an adaptive strategy directing social learners to focus on ecologically relevant behaviours and on tasks that are likely to be learned successfully.

**Supplementary Information:**

The online version contains supplementary material available at 10.1007/s10071-024-01865-7.

## Introduction

Social learning is broadly defined as the process of learning through observation or interaction with others (Heyes 1994). It has been the focus of interest for over a century (Romanes 1884; Morgan 1900; Thorndike 1911), and is well recognized as essential for the acquisition of skills and knowledge (Galef and Giraldeau 2001; Slagsvold and Wiebe 2011; Hoppitt and Laland 2013), and for the development of animal and human culture (Feldman and Cavalli-Sforza 1981; Galef 1992; Creanza et al. 2017; Whiten 2019). However, despite decades of extensive research, our understanding of social learning mechanisms remains incomplete, which make it difficult to integrate mechanistic and functional approaches in the study of social learning and its evolution (Mesoudi et al. 2016; Lotem et al. 2017; Kendal et al. 2018). As a result, ongoing controversies still exist over some fundamental questions, such as the extent to which social learning is adaptive (Rogers 1988; Galef and Giraldeau 2001; Lehmann and Feldman 2009; Arbilly and Laland 2013; Aplin et al. 2017), and whether social learning has evolved as a special adaptation, beyond a flexible expression of domain-general associative learning principles (Heyes and Pearce 2015; Heyes 2016; Lotem et al. 2017; Kendal et al. 2018).

Discussions of these questions have become especially relevant in light of a growing interest in the idea that animals (including humans) have evolved “social learning strategies”, directing them in what, when, and whom they should copy (Laland 2004; Rendell et al. 2010; Smolla et al. 2016; Kendal et al. 2018). The evolution of such strategies is expected because using or not using social learning under different circumstances can have significant positive or negative fitness consequences (Laland 2004; Rieucau et al. 2010; Hämäläinen et al. 2020). Yet, understanding how such strategies can possibly evolve requires to go beyond a simplistic “gene for a strategy” view, and to consider which genetically variable traits control the process of social learning (Lotem and Kolodny 2014; Leadbeater 2015; Mesoudi et al. 2016; Kendal et al. 2018).

It has been suggested that selection for social learning strategies may act on attentional or input mechanisms (Heyes 2012; Leadbeater 2015; Leadbeater and Dawson 2017), on the coordinated action of attentional and learning mechanisms that construct memory representations in the brain (Lotem et al. 2017, 2023), or on mechanisms that assign reinforcement value to social observations (Najar et al. 2020). It is therefore necessary to focus on how such basic mechanisms work, and how their fine-tuning can shape or control the process of social learning. A focus on mechanisms is also necessary for studying the extent to which mechanistic constraints restrict or perhaps mediate (or even facilitate) the evolution of social learning strategies. Simply put, according to the restricting view, animals occasionally fail to learn socially because it is ‘too difficult’ (i.e. the constraints are fixed), while according to the facilitating view, social learning may evolve to be ‘easy’ or ‘difficult’ when it is likely or unlikely to be adaptive (i.e. the constraints themselves are evolving). For both views, however, the mechanistic constraints need to be studied and identified.

There have been several attempts to divide social learning into subcategories that differed in what was assumed to grab learners’ attention and in the role played by demonstrators (Heyes 1994; Hoppitt and Laland 2008). For example, it has been suggested that in the simple case of ‘social facilitation’, the demonstrator hardly demonstrates anything but their sheer presence can improve learning by increasing motivation and reducing neophobia (Zajonc 1965; Dindo et al. 2009; Ward 2012). A greater role has suggested to be played by the demonstrator in ‘stimulus’ or ‘local enhancement’ where highlighting an object or a location is necessary to initiate a successful process of asocial learning (Heyes 1994; Hoppitt and Laland 2008). In ‘observational learning’, it is assumed that the observer pays attention to cues associated with the choices made by the demonstrator (e.g. food-cue association) or to the outcomes of its actions in terms of reward or punishment (Hoppitt and Laland 2008; Truskanov et al. 2020). Finally, in the case of ‘imitation’ or ‘emulation’ the observer is assumed to pay attention to the actions of the demonstrator (e.g. lifting a cover) or to the outcomes of these actions (e.g. the cover has been lifted), and to copy or generate the same actions or consequences (Hoppitt and Laland 2008; Galef 2015; Truskanov and Lotem 2017).

In line with this mechanistic framework, it has been suggested, or implicitly assumed, that some forms of social learning may be cognitively more challenging than others, at least for some animal species (Whiten and Ham 1992; Van Schaik and Burkart 2011). It has also been shown that in some cases animals can fail to learn socially if distracted by scrounging opportunities (Giraldeau and Lefebvre 1987; Beauchamp and Kacelnik 1991). Hence, there are reasons to believe that the expression of social learning, or the use of social learning strategies, may sometime be limited by mechanistic or cognitive constraints. Recently, we proposed that alongside ecological conditions or strategic decisions, a major determinant of the relative use of social as opposed to asocial learning may simply be the relative ease of learning a task with and without social demonstration (Aljadeff et al. 2020; Marković et al. 2023). For that reason, it may be necessary to control for the effects of task-related cognitive demands when testing hypotheses regarding social learning strategies.

For example, we recently found that faced with a task that requires to open covers marked with a rewarding cue, house sparrows that were already pre-trained to open unmarked covers, could use individual experience to learn the most rewarding cues, and their choices could be different than those of the demonstrators (Aljadeff et al. 2020). On the other hand, when the birds were not pre-trained to open the covers, they used social learning for learning both the action of cover opening and the rewarding cues, thereby conforming to the choices made by the demonstrators (Marković et al. 2023). These contrasting results, under otherwise identical conditions, suggested that task-dependent cognitive demands (in this case, having or not having previous experience with cover openings) can strongly affect the use of apparently different learning strategies (i.e., individual learning of the rare alternative as opposed to social conformity). Yet, in order to verify that this is indeed the case, the relative ease of learning to open covers and to choose the rewarding cues, with and without social demonstration, has to be quantified. More generally, in order to control for the possible effect of mechanistic constraints on the expression of social learning strategies (and to further consider the evolution of such constraints), one has to first identify and characterize these constraints.

The goal of the present study was just that. Thus, to assess the effects of mechanistic constraints on the use of social learning strategies, we tested experimentally how different types of social demonstrations improve house sparrows’ (*Passer domesticus*) success in solving a novel foraging task that had two components. The first component required ‘operant learning’: the sparrows had to learn to open paper covers in order to reach millet seeds hidden beneath the covers. The second component of the task required ‘discrimination learning’: the sparrows had to learn to associate the presence of the millet seeds with one of the two colours of the paper covers and to discriminate between them. While these two components, and the social learning task involved, are directly related to our previous studies (Aljadeff et al. 2020; Marković et al. 2023), they are also similar to those used in other studies, and to the challenges that birds are likely to face in nature (Reader and Biro 2010; Riebel et al. 2012; Aplin et al. 2013).

Sparrows in three experimental groups were provided with three different types of social demonstration: Paired demonstration (of both cover opening and colour preference), Action-only demonstration (of opening white covers only), or No demonstration (only a companion bird that forage from exposed wells). We expected sparrows’ success in the No-demonstration group to be poor because in several passerine species, including in house sparrows, learning to open covers has been shown to be difficult without social demonstration or gradual shaping (Aplin et al. 2013; Keynan et al. 2014; Truskanov and Lotem 2017; Aljadeff et al. 2020). On the other hand, we expected that learning to open the covers would occur in both the Paired and the Action-only demonstration groups, because: (a) the action of opening the covers was equally demonstrated in both groups, and (b) it has been shown previously that sparrows can learn to open covers with social demonstration (Truskanov and Lotem 2017). We could also expect that colour preference may be better learned with Paired rather than Action-only demonstration because only in the Paired demonstration the correct choice of colour was demonstrated. However, in many birds, including in sparrows, food-related cues may be learned quite easily without social demonstration (Herborn et al. 2011; Katsnelson et al. 2011; Rojas-Ferrer and Morand-Ferron 2020; Aljadeff and Lotem 2021), suggesting that a demonstration of colour preference may not be needed. In this case no advantage for the Paired over the Action-only demonstration group would be observed. Alternatively, the demonstration of colour preference may increase the probability of being rewarded and may thus interact with the operant learning process, and contribute to its success. Thus, by clarifying how social learning success is affected by both the nature of the learning tasks (operant or discrimination learning) and the kind of social demonstration available to the learner, we hope to better evaluate the role of mechanistic constraints in restricting or mediating the use of social learning strategies.

## Methods

### Study animals and research set-up

The study was conducted at the Meier Segals Garden for Zoological Research, Tel-Aviv University, in August-October 2020 and 2021. We used young house sparrows (*Passer domesticus*) that hatched in the spring of the same year, either hatched in our captive colony (during 2020, N = 60) or in the wild (during 2021, N = 26). At this age it is impossible to visually distinguish between males and females so the sex of the birds that participated in the experiments was unknown. Breeders for the captive colony (see Dor and Lotem 2009), as well as young sparrows caught for the experiments, were captured using mist nets during the early spring or summer of 2020 and 2021 in Hulda and Beit-Kama (central Israel), or at the zoological garden of Tel-Aviv University. The sparrows were initially hosted in large aviaries (each 4 × 4 m and 3 m high, hosting 10–30 individuals in each aviary) and were provided with perching poles and nestboxes for shelter and given a combination of commercial birds' mixture, boiled eggs, cucumbers and water ad libitum.

**Fig. 1:**
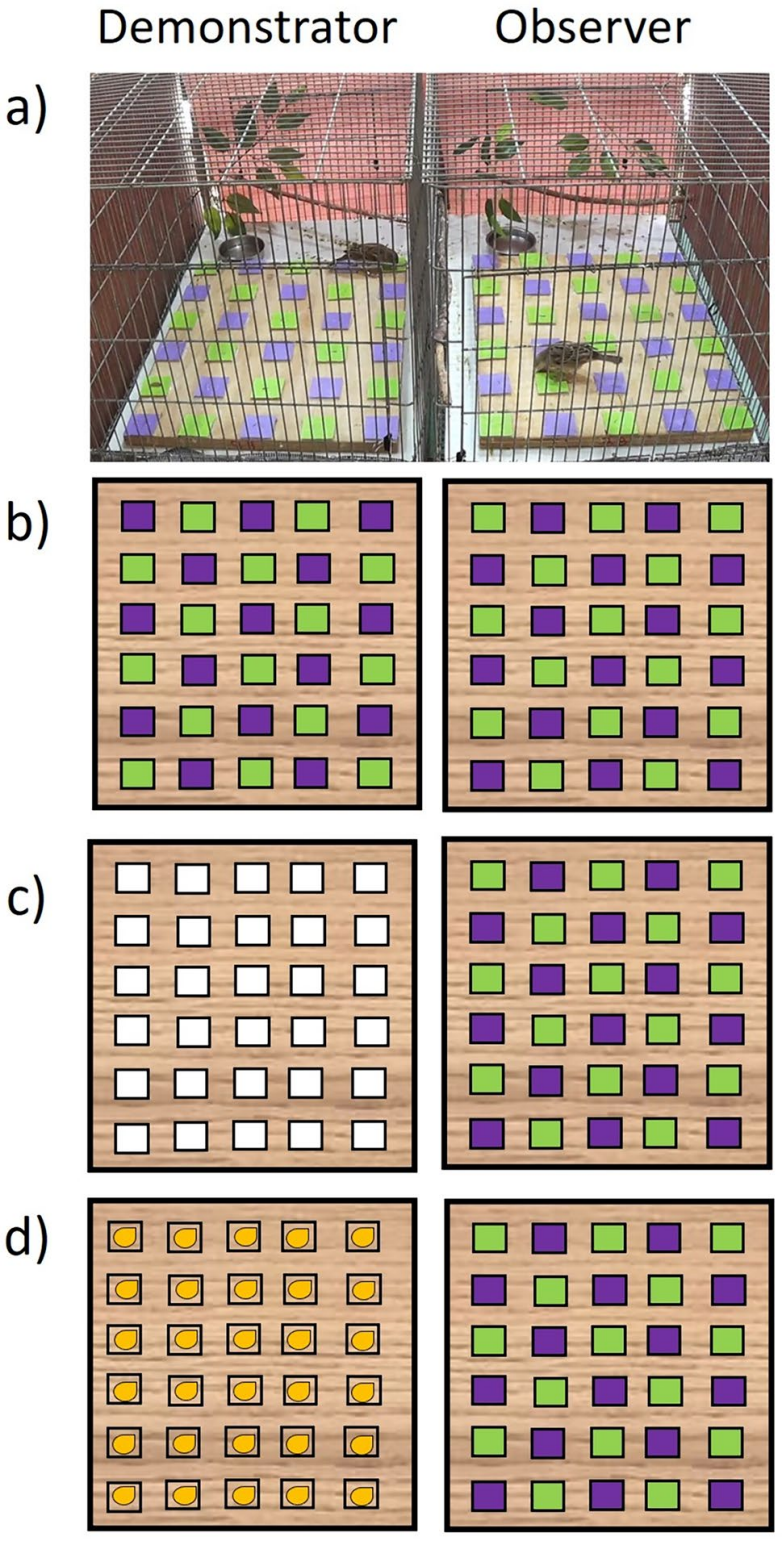
Experimental setup and design: **a** a demonstrator (left) and an observer (right) during a learning session of the Paired demonstration group, **b** Paired demonstration setup in which the demonstrator’s task is nearly the same as the observer’s task, allowing it to provide social motivation as well as to demonstrate both cover opening and colour preference (colour location is not identical to prevent cue learning through location), **c** Action-only demonstration setup in which the demonstrator open white covers, which allows it to provide social motivation and cover opening demonstration, **d** No demonstration setup in which the demonstrator feeds on open wells, which allows it to provide only social motivation (see further explanation in the text)

For the experiments, sparrows from the same aviary were transferred into individual cages (75 $\times$ 45 cm and 45 cm high) that were placed on tables in a shaded outdoor aviary. The cages were positioned side-by-side, creating four sets of paired cages, designed to host an observer and a demonstrator in each pair of cages (see Fig. 1a). The observers and the demonstrators were likely to be familiar to each other as they were taken from the same aviary. To visually isolate each pair of birds from the rest, each pair of cages was blocked from the back and from the sides by plastic sheets, while the top, the front, and the middle joint wall remained uncovered (the joint wall dividing between the observer and the demonstrator was only blocked during specific periods that will be described below). Wooden branches and artificial leaves were positioned at the back of each cage to provide shelter and perching positions. In addition, each cage contained a supply of water and food (seed mixture, graded boiled eggs and cucumber).

During experimental sessions, the food was removed and a wooden foraging grid was inserted through a secured opening located in the front-bottom side of the cage. The grid contained 30 round cavities, hereafter "feeding wells" (2.5 cm diameter, 1.8 cm deep and 8.5 cm distance between wells) that were uniformly spread across it and may or may not contain millet seeds (depending on the treatment regime). Each feeding well could be covered by a square shaped laminated paper (concealing the millet seeds) in different colours (we used green, purple and white in this study). When the well was covered, the seeds could be accessed through a slit in the paper that the sparrows could peck through without removing the paper (see Fig. 1). The green and purple colours provided food-related cues to be learned during the experiments (see below). The described experimental setup in which individual sparrows are placed in individual cages and tested with foraging grids has been used extensively in our laboratory (Katsnelson et al. 2008, 2011; Truskanov and Lotem 2015, 2017; Ben-Oren et al. 2022). We have also used successfully the same paper covers technique described above in two recent studies (Aljadeff et al. 2020; Aljadeff and Lotem 2021).

### Experimental design

#### General structure

Our experiment was based on allowing a single naïve sparrow to forage on a grid while observing a trained demonstrator feeding on a foraging grid in an adjacent cage. We used three types of demonstrations (see below), forming three experimental groups consisting multiple observer-demonstrator pairs. Each observer-demonstrator pair went through an experiment that lasted for 5 consecutive days. Except for the first day, each day included 6–7 sessions that lasted 15 min, with ~ 30 min time intervals between them. Each session included a single presentation of the foraging grid to the sparrow. All the experimental days (days 2–5) began with a 2.5-h food-deprivation period to increase the motivation to later seek food on the grids. On day 1, eight sparrows were captured from the large aviary, transferred to the individual cages, and were randomly assigned to the roles of demonstrators or observers, forming four observer-demonstrator pairs, that were also assigned to one of the three experimental groups (see below). To accustom the birds to the cages and to the foraging grids, they immediately received a grid filled with exposed seeds (i.e. food placed in uncovered feeding wells) and were allowed to forage for 4–5 h. Days 2 and 3 of the experiment were used for training the demonstrators, while days 4 and 5 were used for the social learning sessions.

To train the demonstrators (during days 2 and 3) we used a gradual shaping process that enabled them to acquire the technique of opening the covers. This was done by offering feeding wells with different degrees of cover (from widely open to the final state of a narrow slit). This process was very effective in training all demonstrators to open covers within 2 days, and in the Paired demonstration group, to open covers of the rewarding colour (see below). During these training sessions (6–7 sessions per day) the observer was presented with grids that contained millet seed in exposed wells, and a plastic divider was inserted between the cages, so that the observer could not see the demonstrator and learn from it at this stage. We removed the divider at the end of each session so that the demonstrator and the observer continued to see each other, which helped to reduce stress and increase their familiarity with each other.

Social learning sessions (6 per day) were conducted on Day 4 and 5. At the beginning of each session, the demonstrator received its foraging grid ~ 3 min before the observer, allowing the observer to watch the demonstrator forage on the grid for 3 min after which a grid was also inserted to the observer’s cage. From this stage, the observer could forage on its own grid as well as to continue to observe the demonstrator for the remaining 12 min of the session. For various technical reasons, some birds could not participate (or being recorded) in all 12 social learning session as planned, but only in 11 (*N* = 2), 10 (*N* = 1), or 6 (*N* = 1), which were taken into consideration when analysing the data.

#### The learning task

As mentioned earlier, the learning task presented to the naïve observers required both operant learning: learning the action of opening covers, and discrimination learning: learning which of two colour covers, green or purple, is associated with food. The action of cover opening could be achieved by either pecking through the slit of the cover, by lifting the paper at the edge of the slit, or by a combination of both. The demonstrators were not trained to use only one of these techniques (as in a “two-action” experiment) but could freely learn either one or both techniques during the shaping process (see above). Note that our goal in this study was to test the effect of different types of social demonstration on the sparrows’ ability to open the covers, regardless of the exact social learning mechanism allowing them to do so (e.g., stimulus enhancement, emulation, imitation etc.). Previous work in our laboratory already applied the two-action paradigm to study how sparrows learn to open covers from a demonstrator, showing an effect of social demonstration on the action chosen by the learner, which occurs through a socially mediated trial-and-error process (see Truskanov and Lotem 2017). We believe that the process here is likely to be the same. In the present study, however, we coupled the cover opening task with a discrimination learning task, which was not involved in the previous study. To create the discrimination learning component of the present study, half (15) of the 30 wells of the foraging grid were covered with the rewarding colour (purple or green, randomized across pairs) and the other half with the non-rewarding colour. Covers of the rewarding colour provided food (3 millet seeds) with a probability of 2/3 (in 10 out of the 15 wells, randomly selected) while covers of the non-rewarding colour never provided food (15 empty wells). We used partial reinforcement (10/15 rewarding wells rather than a certain reward probability of 15/15) in order to reduce the effect of extinction when the food is depleted (see Aljadeff et al. 2020; Aljadeff and Lotem 2021; Marković et al. 2023). Sparrows have been shown to discriminate well between green and purple covers, no matter which of the two was the rewarding one (Aljadeff and Lotem 2021). All naïve observers were presented with the described learning task during the social learning sessions (the 12 sessions during days 4 and 5) but were exposed to different type of social demonstration in the three experimental groups.

#### The experimental groups

We compared the effect of three types of social demonstration in three experimental groups: Paired demonstration, Action-only demonstration, and No demonstration. In the Paired demonstration group the demonstrators could demonstrate both the action of cover opening and the preference of the rewarding colour, as they were allowed to forage on a grid with coloured covers (see Fig. 1b). In the Action-only demonstration group, the demonstrators foraged on a grid with white paper covers (see Fig. 1c), so they could only demonstrate the action of cover opening but could not provide information regarding which colour is rewarding (it is possible that the observers could still learn to associate the white colour with receiving food but this could not help in the discrimination learning task). Finally, in the No demonstration group, the demonstrators foraged on a grid without paper covers (Fig. 1d) so they could neither demonstrate covers opening nor colour preference. Nonetheless, they still provided social facilitation or social motivation to forage on the grid, which may still increase the probability of successful learning. Each demonstrator in a pair was trained (during days 2 and 3) for its designated type of demonstration. Demonstrators in the Paired demonstration group were gradually shaped to open covers of the rewarding colour that provided three seeds with a probability of 2/3 (as in the target learning task of the observers). Action-only demonstrators were shaped to open white covers of wells that always contained one seed. Demonstrators of the No demonstration group were trained to forage on a grid with exposed feeding wells that contained one seed in each well. Note that the overall amount of food provided on the grid was the same in all groups (10 × 3 seeds = 30 × 1 seed), aiming to minimize variation in hunger and motivation between the different types of demonstrators. The difference in the proportion of wells that contained seeds in the Paired and the Action-only groups (2/3 versus 1) was unlikely to affect the perceived number of cover opening demonstrations with and without food extraction because the demonstrators of both groups revisited and opened wells multiple times (up to 100 or more per grid per session, see Results). These repeated visits added many demonstrations of cover openings without food extraction which greatly diluted the initial differences in the proportion of reward between groups. Moreover, not all 3 seeds in the Paired group were necessarily extracted in a single visit, making the overall proportion of food extractions exhibited by the two types of demonstrators quite similar. The possibility that some remaining differences can still explain our results will be addressed in the Discussion section.

We initially carried out the experiment with 10 observer-demonstrator pairs of each experimental group (60 birds in total) during August-October of 2020. To allow better examination of some of the unexpected differences found between Action-only and Paired demonstration, we run additional experiments during August-October 2021, with 13 additional observer-demonstrator pairs, six with Paired demonstration, and seven with Action-only demonstration, thus increasing the sample size of these treatment groups to N = 16 and 17, respectively. The results of the two sets of experiments (of 2020 and 2021) were similar qualitatively (see Fig. S1) as well as quantitatively (see Statistical analysis sub-section below and Table S3) and were therefore combined for the main statistical analysis.

### Behavioural and data analysis

All shaping and social learning sessions were recorded using HD video cameras that were positioned outside the cages and allowed detailed behavioural analysis of observers and demonstrators. Video analysis was carried out using a Python-based software (*Poke-a-bird 0.7*, http://arnonlotem.weebly.com/technical-tools--code.html) developed by Michal Keren. Each sparrow was analysed separately, and the following distinct events were scored with their time and location (i.e. the identity of the visited feeding well): landing on the grid, leaving the grid, ‘peck’—inserting the beak through the slit in paper cover (or after tearing the cover), and ‘attempt’- any interaction with the cover that did not involve a peck (such as pulling the sides or the corners of the cover without opening it). We used the time of first landing on the foraging grid during the learning stage to assess the bird’s level of neophobia or fear, which can potentially affect learning. To that aim we scored the chronological number of the session of first landing (from 1 to 12), and the latency to first landing in seconds, from the beginning of the first session (latencies for birds who first landed on session 2 were calculated by adding the duration of session 1; no bird had its first landing after session 2, see Results).

We distinguished between two types of pecking events: pecks in intact wells and pecks in previously opened wells (birds often returned to the same wells). Intact wells were those that were pecked for the first time during a learning session. Wells that have been pecked previously (i.e. not intact) tend to appear open or torn, possibly providing indication of reward or past choices that are unrelated to the colour cue, and may be much easier to open. Therefore, only pecks in intact wells were used to measure the learning success of the observers. The success of operant (action) learning was measured by: (a) the speed of learning measured as the chronological number of the session of first cover opening (i.e., 1 for birds that first opened in the first session, 2 for birds that first opened in the second etc., and the total number of sessions + 1 (i.e., 13 or 12) for birds that never opened wells, representing the minimal time it would have taken them to learn if the experiment was longer), (b) the mean number of pecks (opening of intact wells) per session (calculated as: total number of pecks in intact wells/total number of sessions during the learning stage), and (c) the mean number of pecks per active sessions, which is the same as ‘b’ but uses only the number of sessions during which the bird already knew to open covers, thus measuring how frequently the learner used the learned action regardless of how long it took to learn it [calculated as: total number of pecks in intact wells/(total number of sessions ˗ number of sessions before first opening)].

The success of discrimination learning was measured as the proportion of pecks in intact wells of the rewarding colour. We used only the first 15 intact wells visited in a session for this analysis (or less, if less than 15 intact wells were visited) because later choices may be biased by the lack of available intact wells of the preferred colour (only 15/30 wells on a grid were of the rewarding colour). Most pecks in intact wells that contained seeds (10 out of 15) led to successful feeding but it was not always possible to verify it from the videos. However, we can still view a peck in an intact well that contained seeds as a peck with a high probability of extracting seeds and thus as being rewarded for the action and for the choice of colour.

Although all demonstrators learned and performed the task they had to demonstrate (including the demonstrators of the paired group that opened covers of the rewarding colour in 80–100% of their demonstrations), their level of activity during the social learning sessions varied, which may have affected the number of learning opportunities given to their observers (Van Leeuwen et al. 2021). Therefore, a demonstrator’s level of activity during the social learning stage (mean number of pecks per session in exposed wells in the No demonstration group, and mean number of pecks per session in the Action-only and Paired demonstration groups) was included in the statistical analysis as an additional explanatory variable of learning success. Note that for this analysis we used all types of pecks, not only pecks in intact wells (as we did for measuring the learning success of the observers), and not only pecks in rewarding wells. This is because all types of pecks performed by the demonstrator could be seen by the observer and affect its ability to learn the action of cover opening.

To assess whether the observers were at all interested in the demonstrators’ cages, and were not avoiding them, we scored each observer’s location in the cage during the three minutes after the demonstrator received their foraging grid and while the observer was still waiting to receive its own (see above). It was quite apparent that at this stage, most observers were interested in the demonstrators and/or in their newly introduced foraging grid, but because birds’ eye gaze is difficult to measure we used their tendency to get closer to the demonstrator as a possible proxy for their social interest. The location at the cage was determined after dividing the space of the cage (using the video analysis software) into four quarters representing the level of proximity to the demonstrator (quarter 4 as the closest, see Fig. S2). We sampled birds’ location at the cage every 5 s during the 3 min of waiting (36 samples) in sessions 1, 2 and 7, 8 of the social learning stage (which were the first two sessions of each day of the learning stage). The mean of these 36 samples was taken as the observer’s proximity score for that particular session. It should be noted that while the proximity score may indicate general interest in the demonstrator or in the foraging grid, it cannot tell us whether the observers paid attention to the demonstrators’ particular actions or choices.

### Statistical analysis

Statistical analyses were conducted using R version 4.0.4 (R Core Team 2022). We analyzed the results using generalized linear models where applicable (GLM with a Poisson or quasi Poisson distribution, function glm() from the {stats} package in R language, see further details below), and nonparametric tests, otherwise (Spearman rank correlation coefficient (r_s)_ for correlations, Kruskal–Wallis rank sum tests, Mann–Whitney U tests, or Wilcoxon rank sum tests, for comparing independent groups, and Binomial tests for testing whether proportion of choices was different than expected by chance). Reported P values of the GLMs were extracted using the function Anova() from the R car package. All reported P values are two tailed. We used the generalized linear models (GLM) to test the extent to which differences between experimental groups were affected by variation in demonstrators’ level of activity (see above). Accordingly, the models tested the effect of experimental group (as a categorical variable), the number of demonstrations (as a continuous variable), and the interaction between them, on the three different measures of social learning success (speed of learning, mean number of pecks per session, and mean number of pecks per active sessions). Because almost no learning took place in the “No demonstration group” (see Results), this group was not included in the model where learning success was measured by the number of pecks during active sessions (as there were almost no active sessions in this group). To test the differences between the Paired and the Action-only groups separately, we also ran the other two models (where learning success was measured by speed of learning and by the mean number of pecks per sessions) without the “No demonstration” group (see Results and Table 1 therein for the five final models).

Although the interaction between experimental group and number of demonstrations was not always significant, it was always indicated visually in the figures, suggesting that the lack of significance may be due to low statistical power. In such cases, removing the interaction term may weaken the main effects and may not be justified. We therefore kept the interaction term in all five models presented in the main text, but additionally provided the results of the three relevant models after removing the non-significant interaction terms (see Table S1), showing that the main results are still statistically significant, though somewhat weaker, as expected.

We also ran the five statistical models while including the latency to land on the foraging grid (see above) as an additional continuous predictor. The effect of latency was never significant and its inclusion in the models did not change the main results (see Table S2). Finally, to verify that the data sets of 2020 and 2021 that look similar (Fig. S1) can be combined for the main statistical analysis, we ran the three statistical models comparing the Paired and the Action-only groups (for which there was data from both years) while including year as a categorical factor, as well as year and its interaction with experimental group. The effects of year and its interaction with group were never significant and their inclusion in the model did not change the main results (see Table S3).

## Results

### Latency to approach the foraging grid

Except for two birds (from the No demonstration group) who made their first landing on the foraging grid during the second session, all other observers made their first landing on the foraging grid during the first session of the learning stage (see Fig. S3a). After the first landing, all birds made repeated landings and spent time on the grid during the remaining sessions (up to the 12th session), suggesting that their ability to learn to open covers was not limited by fear or neophobia that restricted their presence on the grid. We further analysed the observers’ latency to first landing on the grid (measured in seconds from the beginning of the first session), showing that there was no difference in latency between the Paired and the Action-only demonstration groups (Fig. S3b; Mann–Whitney U test, *P* = 0.603, N = 16, 17, respectively). Yet, the latency of the No demonstration group was significantly longer than that of the Paired and Action-only groups combined (Fig. S3b; Mann–Whitney U test, *z* = 2.08, *P* =  < 0.05, N = 9, 33, respectively). This result suggests that facing the coloured covers for the first time, the sparrows possibly experienced some initial fear, but this fear was reduced if they had a demonstrator that also faced covers and willingly approached them, and regardless of whether these covers were white (Action-only group) or coloured (Paired group). Finally, although latencies were relatively short (in comparison to the length of the learning period), to examine whether variation in latency could still affect our experimental results we re-ran the main GLM models (that will be presented below), while including latency as an additional continuous predictor. The effect of latency was never significant (Table S2), and forcing it into the model did not change the main results (compare Table S2 with Table 1 below).

**Table 1:** Statistical GLM models testing the effect of experimental group and number of demonstrations on three different measures of social learning success (models A and C includes all three groups, models B, D, and E includes only the Paired and Action-only groups)*

Model	*N*	Response variable	Fixed effects	*df*	χ2	*P*
A	41	Session of first opening	Experimental group	2	46.394	** < 0.001**
			Number of demonstrations	1	0.134	0.715
			Interaction	2	8.816	**0.012**
B	32	Session of first opening	Experimental group Number of demonstrations Interaction	1 1 1	16.515 0.12 8.788	** < 0.001** 0.729 **0.003**
C	41	Mean number of pecks per session	Experimental group	2	12.154	**0.002**
			Number of demonstrations	1	7.215	**0.007**
			Interaction	2	2.625	0.269
D	32	Mean number of pecks per session	Experimental group	1	5.724	**0.017**
			Number of demonstrations	1	5.787	**0.016**
			Interaction	1	2.102	0.147
E	24	Mean number of pecks per active sessions	Experimental group	1	2.887	0.089
			Number of demonstrations	1	4.664	**0.031**
			Interaction	1	1.454	0.228

* We used Poisson distribution for models A and B, and Quasi-Poisson distribution for models C, D and E

### Operant learning: the effect of the type of demonstration

**Fig. 2:**
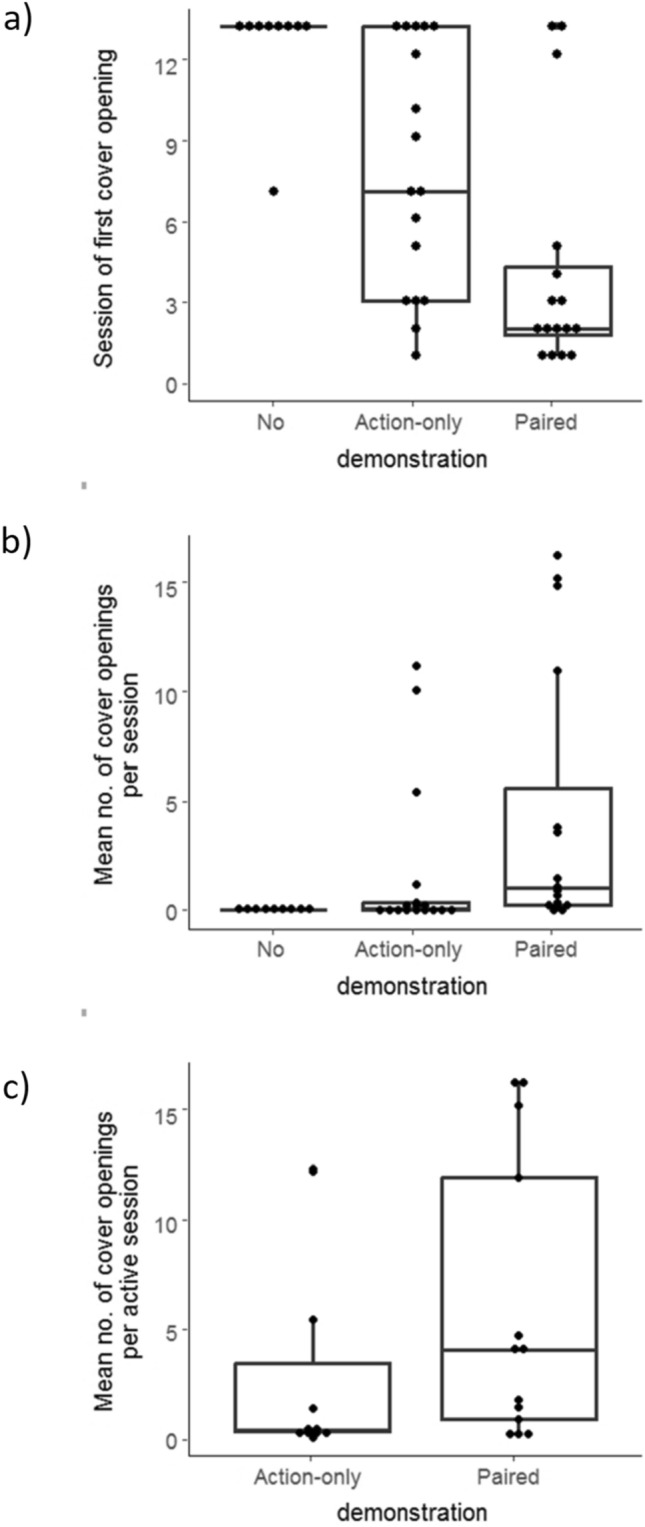
Observers’ success in learning the action of cover opening as measured by **a** the session of first cover opening, **b** mean number of cover openings (pecks) per session, and **c** mean number of cover openings per active sessions (see text for more details). Boxplots show the median and 25th and 75th percentiles; the whiskers indicate the values within 1.5 times the interquartile range, and dots are the data points

The success of naïve observers in learning the action of cover opening when exposed to the three types of social demonstration is described in Fig. 2. Only one of the 10 learners in the No-demonstration group managed to open multiple covers, but as revealed through the video analysis, this was a result of a human error in the grid setup (during the third session) that caused one of the wells of the rewarding colour to bend and to expose the millet seeds. This allowed the bird to detect and approach the exposed seeds and consequently to learn to open wells afterwards (similar to the shaping process we use for training the demonstrators). Excluding this bird from the analysis, only one of the nine remaining birds in the No demonstration group succeeded to open a cover once, as opposed to 11/17 and 14/16 in the Action-only and Paired demonstration groups, respectively, that succeeded to open covers, mostly more than once (Fig. 2a, b).

The birds from the three demonstration groups differed significantly in their speed of learning (Fig. 2a; Kruskal–Wallis rank sum test, *χ*^*2*^ = 16.7, *df* = 2, *N* = 42,* P* < 0.001). Dunn’s multiple comparisons (adjusted with the Benjamini–Hochberg method) show that all groups differed significantly from each other (with *P* < 0.05). Thus, the effect was not only due to the fact that most birds in the No demonstration group never learned (and scored the maximum number of sessions + 1), but also due to birds in the Paired demonstration group being faster than in the Action-only demonstration group (Fig. 2a; *z* = 2.242, *N* = 16,17, respectively, *P *_*adjusted*_ = 0.037). The three experimental groups also differed significantly in the mean number of cover openings per session (Fig. 2b; Kruskal–Wallis rank sum test, *χ*^*2*^ = 15.6, *df* = 2, *N* = 42,* P* < 0.001). In this case as well, all group differences were significant (Dunn’s multiple comparison, *P* < 0.05), implying that birds from the Paired demonstration group opened more feeding wells than birds from the Action-only demonstration group (Fig. 2b; *z* = 2.128, *N* = 16,17, respectively, *P *_*adjusted*_ = 0.033). Finally, comparing the Action-only and Paired demonstration groups, where most of the birds learned to open covers, the mean number of cover openings per active sessions appeared to be higher for the Paired demonstration group, but not significantly so (Fig. 2c; Wilcoxon rank sum test, W = 54, *N* = 14,11, *P* = 0.217).

### Operant learning: the effect of the number of demonstrations

**Fig. 3:**
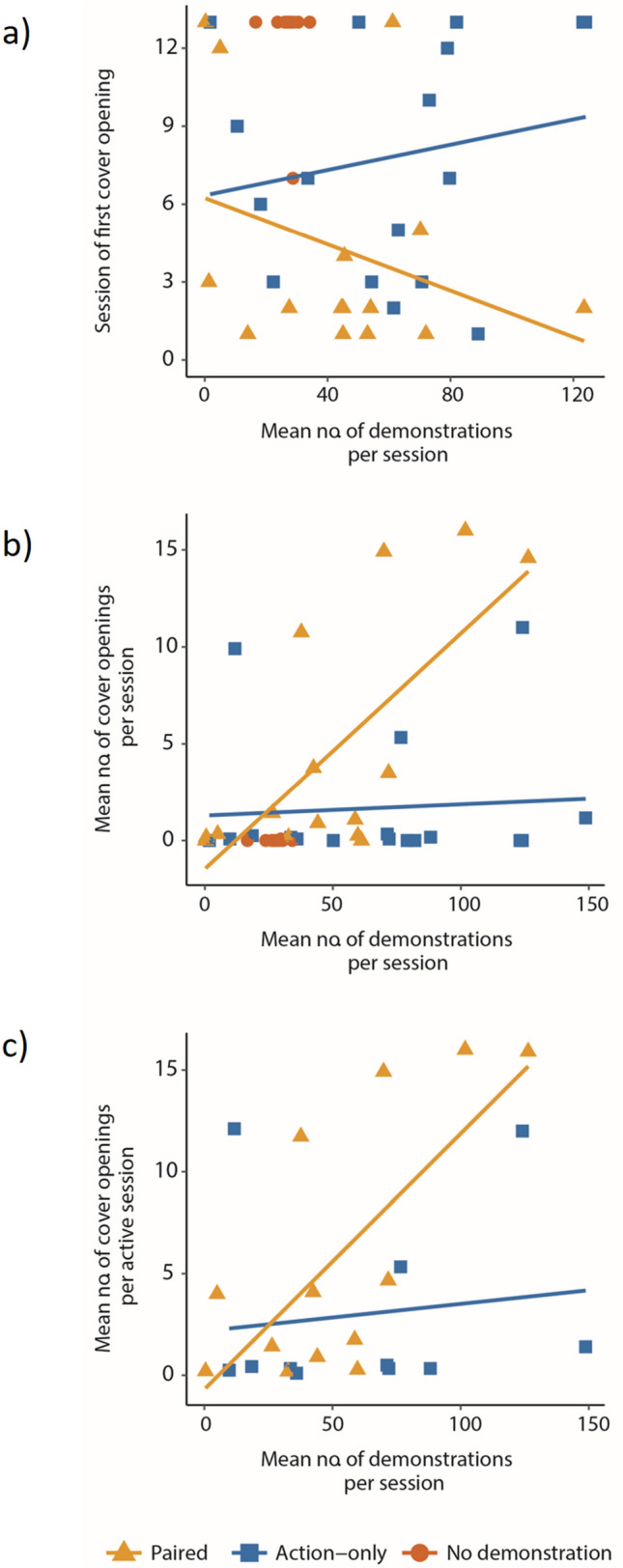
Success in learning the action of cover opening by observers from the Paired (triangles), Action-only (squares), and No (circles) demonstration groups, in relation to the number of demonstrations per session, using three measurements of learning success: **a** the session of first cover opening, **b** mean number of cover openings (pecks) per session, and **c** mean number of cover openings per active sessions (the No group is not shown in this panel because almost all birds in this group failed to learn to open covers and therefore had no active sessions). Trend lines are illustrated only for the Paired (triangles) and Action-only (squares) demonstration groups (see statistics in Table 1 and in the text)

The combined effect of experimental group and the number of demonstrations performed by the demonstrator is illustrated in Fig. 3 and analysed in Table 1 (Models A-E). Recall that the number of demonstrations includes all types of pecks that could affect the learner, including repeated pecks at the same well (see Methods). One observer-demonstrator pair from the Paired demonstration group, for which only six learning sessions could be carried out, was removed from this analysis, reducing the sample size of that group to *N* = 15.

To analyse the factors effecting the speed of learning (Models A and B) we used the chronological number of the session of first cover opening as the dependent variable (i.e. 1 for birds that first opened in the first session, 2 for birds that first opened in the second etc., and the total number of sessions + 1 (i.e. 13 or 12) for birds that never opened wells, see Methods). We then included in the model the mean number of demonstrations per session, calculated based on all the sessions before and during the first opening session (i.e. all demonstrations that were likely to affect the speed of learning). The results (Fig. 3a; Table 1, Models A and B) showed a highly significant group effect (thus confirming the group effect shown earlier in Fig. 2a), as well as a significant interaction between experimental group and number of demonstrations, suggesting that the number of demonstrations improved the speed of learning only in the Paired demonstration group (see Fig. 3a). Note that these results still hold also after removing the No demonstration group from the analysis (Model B).

To analyse the factors affecting learning success as measured by the mean number of openings per session we calculated the mean number based on all sessions (including those before the first opening where the number of openings was zero), and correspondingly, the mean number of demonstrations based of all sessions (since all of them could affect the overall number of openings by the observers). The results (Fig. 3b; Table 1, Models C and D) showed a clearly significant group effect (confirming the group effect shown earlier in Fig. 2b), a significant effect of the number of demonstrations, but no significant interaction (Table 1, Models C and D). Yet, although the interaction was not significant, Fig. 3b suggests that such an interaction may still exist as the positive effect of the number of demonstrations appeared stronger in the case of the Paired demonstration group (*r*_s_ = 0.6, *P* = 0.018) than in the Action-only demonstration group (*r*_s_ = 0.055, *P* = 0.833).

Finally, Fig. 3c and Model E of Table 1 show the analysis of learning success as measured by the mean number of openings per active sessions (the frequency of using the action after it was learned—see Methods). The results of this analysis showed a significant positive effect of the mean number of demonstrations per session on the mean number of wells opened during active sessions, but no significant group effect (see Model E of Table 1). However, a non-significant trend, similar to the one observed in Fig. 2c, is also indicated here, suggesting a slightly higher number of openings during active sessions in the Paired demonstration group. This trend is also supported by the fact that the number of openings during active sessions (in the Paired and Action-only groups) was positively related to the speed of learning (*r*_s_ = 0.446, *N* = 24, *P* = 0.029), which was higher in the Paired demonstration group (Fig. 2a).

While the statistical models controlled for variation in the number of demonstrations, we should note that this number was similar in the Action-only and the Paired demonstration groups (Fig. 3b; Wilcoxon rank sum test, *W* = 163, *P* = 0.189), but was somewhat different among all three groups as a result of a lower number of demonstrations in the No demonstrations group (Fig. 3b; Kruskal–Wallis, χ^2^ = 6.61, *df* = 2, *P* = 0.0367). The reason for this was clear: in the No demonstration group, the wells were not covered so the demonstrators could easily consume the seeds after 20–40 pecks and notice that the wells are empty after that. Yet, 20–40 demonstrations were clearly sufficient for successful learning by quite a few birds in the Paired and Action-only demonstration group (see Fig. 3a), suggesting that in line with the statistical analysis (Table 1, Models A and C), the sparrows’ inability to learn in the No demonstration group was not due to a limited number of demonstrations.

### Discrimination learning

**Fig. 4:**
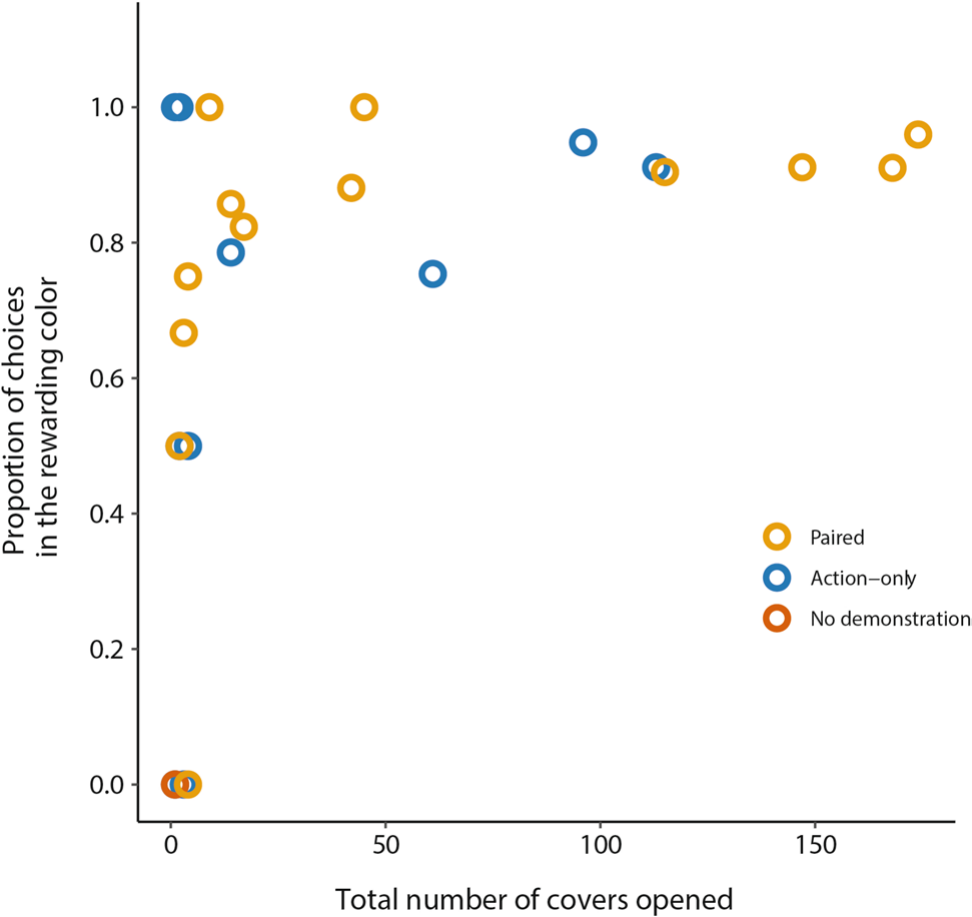
The proportion of choosing the rewarding colour (by observers that learned to open covers from all experimental groups) in relation to the total number of covers opened during the entire learning stage. The proportion of choosing the rewarding colour was calculated based on the first 15 pecks of each session (see Methods), summed up for the entire learning stage

Among the observers that learned to open covers, the proportion of choosing the rewarding colour was positively related to the number of cover openings (Fig. 4; *r*_s_ = 0.37, *N* = 26, *P* = 0.065). This trend is expected by the effect of experience on colour preference, as well as by the effect of correct choices on the tendency to peck more (or by a combination of both). More importantly, all 13 birds that opened at least 5 wells, including four birds from the Action-only demonstration group (that had no colour demonstration), showed a significant preference for the rewarding colour (see Table S4). These results suggest that colour demonstration was not necessary for learning colour-food association, which is consistent with previous work (see Discussion). There was also no indication that the colour of the rewarding cover affected discrimination learning (Table S4; mean choice proportion of rewarding green and rewarding purple was 0.9 and 0.88, respectively, Mann–Whitney U test, p = 0.77).

To assess the effect of colour demonstration before it could be confounded by self-experience, we analysed the number of opening ‘attempts’ (see Methods) that were made by the learners of the Paired demonstration group at each colour before their first successful cover openings. The results (Table S5) show that most birds performed more attempts at the demonstrated colour (Binomial test, 10/13, 2 ties, *P* = 0.048), suggesting an observational learning effect. A similar analysis of the number of opening attempts in the Action-only group, where no colour demonstration was provided, shows no significant preference for the rewarding colour (Binomial test, 10/16, 1 tie, *P* = 0,226).

### Observers’ proximity to the demonstrators

**Fig. 5:**
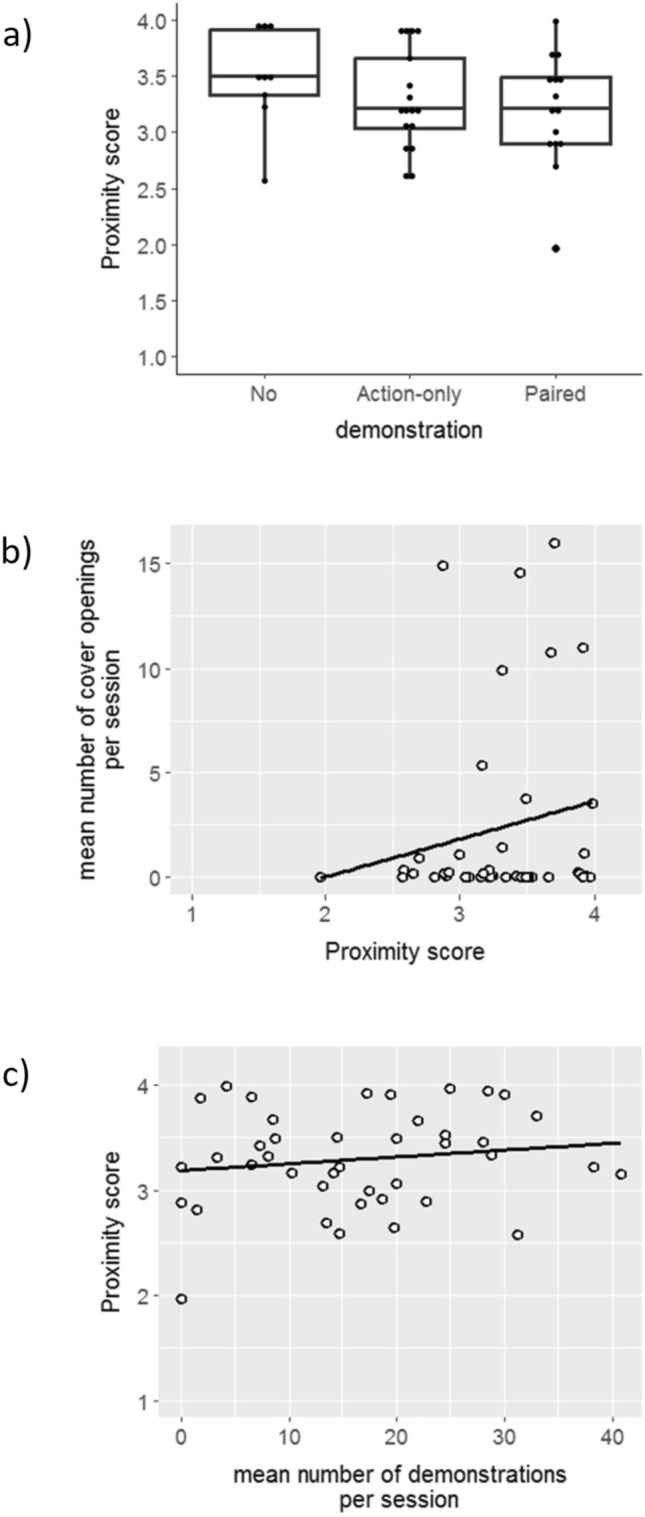
Observers’ mean proximity scores in relation to **a** experimental group (Boxplots show median and 25th and 75th percentiles; the whiskers indicate the values within 1.5 times the interquartile range, and dots are the data points), **b** mean number of cover openings per session, and **c** mean number of demonstrations per session (see statistics in the text)

The proximity score of the observers (in a scale of 1 to 4, where 4 is the closest to the demonstrator’s cage, see Methods) did not differ between experimental groups (Fig. 5a; Kruskal–Wallis rank sum test, *χ*^*2*^ = 3.083, *df* = 2, *N* = 41,* P* = 0.214) and was clearly higher than expected under the null hypothesis of random location at the cage (Fig. 5a; 40/41 above 2.5 score, Binomial test P < 0.0001). Thus, the observers were clearly getting closer to the demonstrators as soon as the foraging grid was inserted to the demonstrators’ cages. For sparrows of the Action-only and the Paired demonstration groups (conditions that enabled social learning) there was a trend suggesting that the proximity score of an observer is positively related to its social learning success (Fig. 5b; *r*_s_ = 0.32, *N* = 32, *P* = 0.074), but not to the activity of its demonstrator (Fig. 5c; *r*_s_ = 0.0031, *N* = 32, *P* = 0.865).

## Discussion

The results of our study show that sparrows were unlikely to learn the action of cover opening without social demonstration (or without gradual shaping as provided to the demonstrators). However, as soon as they learn this action socially (in the Action-only and Paired demonstration groups) they can easily learn to prefer covers of the rewarding colour even without social demonstration (i.e. in the Action-only demonstration group where no colour preference was demonstrated). Interestingly, and in contrast to our predictions, the action of cover opening was learned faster with Paired rather than Action-only demonstration, despite being equally demonstrated in both. In the following we discuss our results, first in respect to the importance of social demonstration for learning novel actions and food-cue associations, and second, in the broader context of how such cognitive processes may shape social learning strategies and their evolution.

### Social demonstration and operant learning

The results show that in all three experimental groups, the observers approached the foraging grid right from the beginning of the learning stage (Fig. S3), and preferentially spent time closer to the demonstrator’s cage (Fig. 5a). Thus, in all groups the observers seemed to notice the presence of the demonstrator and/or the presence of its foraging grid, and had multiple sessions during which they could observe the demonstrator and interact with their own foraging grid. However, in the No demonstration group, observing the demonstrators that merely foraged from exposed wells and could not demonstrate the action of cover opening was evidently insufficient to allow successful learning of this action. Only one of the nine birds of this group opened a single cover (at the seventh session) and did it only once. As noted earlier, this poor learning by the No demonstration group could not be explained by an insufficient number of observations (see Fig. 3 and statistical analysis in Table 1) but rather by the fact that the action itself was not demonstrated. We can therefore infer that the successful learning of cover opening by sparrows in the Action-only and Paired demonstration groups was indeed due to observational learning of the demonstrated action. It is still possible that with a longer learning period and with more demonstrations of other birds eating from exposed wells (i.e. more social facilitation), the action of cover opening could have been learnt also under the No demonstration condition. However, the fact that it did not happen under the experimental conditions suggests that solving the task of cover opening without social demonstration or gradual shaping (as we trained the demonstrators) is indeed difficult.

Learning to open covers without social demonstration (or gradual shaping) has been shown to be difficult for several passerine species, including house sparrows (Aplin et al. 2013; Keynan et al. 2014; Truskanov and Lotem 2017; Aljadeff et al. 2020). This also seems to be the case for similar tasks that requires the learning of novel actions, such as opening a sliding door of a puzzle box by great tits (Aplin et al. 2015), or lifting the lid of a garbage bin by sulphur-crested cockatoos (Klump et al. 2021). In all of these cases it seems that the required novel actions, or sequences of actions, were much more likely to be performed with social demonstration than without it. The tendency to rely on social rather than individual learning in such cases may simply reflect the fact that it is much easier to learn these actions socially rather than individually (but see further discussion in the last sub-section below).

### Why was operant social learning improved when colour preference was also demonstrated?

Although the action of covers opening was equally demonstrated in the Action-only and the Paired demonstration groups, surprisingly, operant learning was significantly faster in the Paired demonstration group (Figs. 2a, 3a, and Models A, B in Table 1). It is important to note that the speed of operant learning was determined by the number of learning sessions required to reach the first cover opening, which occurs before the learner can gain any feedback regarding which of the two cover colours is the rewarding one. This point is important because although knowing to choose the rewarding colour can further reinforce the action of cover opening (since more openings yields food finding), it can do so only after the first opening, not before it. Therefore, even if birds in the Paired demonstration group learned the rewarding colour by observing the demonstrator (see below), this information could not help them to learn to open the first cover faster than the birds of the Action-only demonstration group (up to this point, finding or not finding food under the cover have not yet happened). For the same reason, this result can also not be explained by a contrast between the observed success rates (of the demonstrators) and the ones experienced by the learners (a contrast that could have been slightly greater in the Action-only demonstration where a single seed was provided in all wells, as opposed to the Paired group where 3 seeds were provided in 2/3 of the rewarding wells). If anything, the only difference that learners could potentially see before their first well opening is the slightly higher success rate of the Action-only demonstrators, which would have accelerated learning in the Action-only group rather than vice-versa. Thus, the faster learning of the Paired demonstration group requires another explanation that is not related to the probability of the action or the demonstrations being rewarded.

We suggest that the greater effectiveness of the Paired demonstration may be explained by the greater similarity between the demonstrated and the learned task (see Fig. 1), which may increase the learner’s attention to the demonstrator’s behaviour. This interpretation can also explain why the speed of learning and the number of cover openings were related to the demonstrator’s level of activity in the Paired but not in the Action-only demonstration group (Fig. 3, Table 1). When learners pay attention to the demonstrator behaviour, it is expected that the more cover openings it demonstrates, the more likely that the learners learn the task. On the other hand, if attention to the demonstrator is sporadic, or not directed at its actions, which might have been the case in the Action-only demonstration group, the probability of successful learning should be lower, and the relationship with the number of demonstrations may not be clear, which fits our results (Figs. 2 and 3). An alternative explanation for the effect of task similarity, rather than greater attention to similar tasks, is to assume equal attention but greater tendency to execute the observed behaviour when the task is similar. Both mechanisms lead to the same outcomes and are therefore difficult to tease apart (see (Chimento et al. 2022) for a recent discussion).

The role of perceptual and contextual similarity in social learning has been studied in animals in relation to the similarity between the learner and the demonstrator (Monfardini et al. 2014) or in respect to the learner’s ability to generalize from the learned context to different ones (Root-Bernstein 2010; Truskanov et al. 2018; Arbon et al. 2023). In human psychology there has been some interest in how perceptual and contextual similarity affect skill learning and social learning (e.g. Solomon 1977; Wifall et al. 2014). However, to the best of our knowledge, the role of the similarity between the demonstrated and the learned task has been largely ignored in the literature of animal social learning (e.g. Galef and Giraldeau 2001; Laland 2004; Hoppitt and Laland 2013; Kendal et al. 2018). An interesting parallel to the possible effect of the similarity between the demonstrated and the learned task is the effect of the physical distance between the two. It has been shown, for example, that starlings’ response to social information diminishes with the distance from the neighbouring bird (Fernández-Juricic and Kacelnik 2004). Both physical distance and task similarity may indicate the extent to which the information is relevant. In the case of physical distance, spatial correlation of food density implies that increasing foraging efforts may be justified after seeing another birds finding food nearby but not at a distance (Fernández-Juricic and Kacelnik 2004). In a similar manner, trying to imitate an individual dealing with a task that appears different than the one faced by the learner may be futile. Thus, the suggested effect of task similarity may not reflect a mechanistic constraint but rather a learning strategy (see further discussion below).

Finally, the greater effectiveness of the Paired demonstration group is also suggested by the number of cover openings during active sessions. That is, birds of the Paired demonstration group tended to open more covers per active sessions (Figs. 2c, 3c, and Model E of Table 1), and birds that were fast in learning to open their first cover, also opened more covers per session after that (there was a significant positive correlation between speed of learning and pecks per active sessions). There are two, not mutually exclusive, explanations for this result. First, fast learning may be associated with greater motivation, confidence and proficiency, which could also result in greater cover openings activity (which is consistent with the positive correlation found between speed of learning and the number of pecks per active sessions). Second, it is possible that the effect of the demonstrators’ level of activity that resulted in faster learning also affected the learners’ level of activity after the first cover opening. In other words, watching active demonstrators encouraged the learners to increase their own foraging activity.

### Social demonstration and discrimination learning

The analysis of cover opening attempts shows that the sparrows in the Paired demonstration group tended to direct their attempts to the demonstrated colour even before their first successful opening attempt. This result provides clear indication that house sparrows can learn to discriminate between colours by observing others. Various forms of observational learning have been reported in passerine species (e.g. Fritz and Kotrschal 1999; Emery and Clayton 2001; Hoppitt and Laland 2008; Riebel et al. 2012; Aplin et al. 2013; Brodin and Utku Urhan 2014), as well as in our house sparrow population (Truskanov and Lotem 2015, 2017). The present finding adds to these studies by clearly showing a case of observational learning of food-cue association and may suggest that social demonstration of colour preference can potentially improve discrimination learning. However, as soon as the sparrows in our study learned to open covers repeatedly, all of them learned to prefer the rewarding colour either with or without colour demonstration (i.e. in both the Paired and the Action-only demonstration groups, see Table S1). There is no indication therefore (at least in our data) that observational learning in sparrows can improve or accelerate discrimination learning. The reason for this is probably that sparrows can easily and rapidly learn food-colour association through individual learning, which has been shown in our previous studies (Katsnelson et al. 2011; Ilan et al. 2013; Truskanov et al. 2018; Aljadeff and Lotem 2021; Ben-Oren et al. 2022; Marković et al. 2023).

Our study suggests that social demonstration of colour preference does not improve the learning of food-colour association when social and private information are consistent with each other. Yet, we did not examine the case of conflicting information which allows to assess the relative weight given to social as opposed to individual information. Previous work suggests that in the presence of conflicting information sparrows rely much more heavily on their personal experience (Ilan et al. 2013; Truskanov and Lotem 2015). However, recent work in our lab suggests that when the learning task is made more difficult by requiring operant learning in addition to the discrimination learning task, sparrows may rely more heavily on social learning, even when doing so is suboptimal (Marković et al. 2023). This recent work, as well as the present study, show that while it is generally true that, for house sparrows, food-cue association is easy to learn individually and operant learning is easier to learn socially, the interaction between these two tasks may lead to non-trivial results. In Markovic et al. (2023) operant learning affected the learning of food-cue association, and in the present study the demonstration of a similar task in respect to discrimination learning affected operant learning.

### Social learning strategies and the relative ease of learning a task socially as opposed to individually

One of the goals of our study was to evaluate how the relative ease of learning a task socially as opposed to individually can shape the expression of social learning strategies. We also mentioned that identifying mechanistic constraints that make one type of learning easier than the other may not imply that these constraints are fixed or inevitable. It is possible that these constraints themselves have evolved to tune the adaptive expression of social learning strategies (see Introduction). In line with previous work, the results of our study show that food-cue associations are easy to learn individually while novel actions are easier to learn socially. There are probably some objective reasons that make operant learning difficult to learn individually rather than socially. For example, solving a task that requires a specific action (or set of actions) out of many possible ones, implies a large search space that can be narrowed down significantly by social demonstration. On the other hand, using an already learned or natural action (e.g. pecking) to explore the reward value of alternative cues is easy to do individually. Yet, our results also show that operant learning was more difficult when it was not paired with a demonstration of food-colour association, which cannot be explained by objective task difficulties (as these were the same in the Action-only and Paired demonstration). The idea that task similarity increased learners’ attention to the demonstrated behaviour (as suggested above) implies an adaptive mechanism of tuning attention to relevant information. In other words, evolving social attention more broadly should have been possible (i.e. it is not more ‘difficult’) but was probably selected against if resulted in learning behaviours that are out of context and are therefore unlikely to be useful or successful. This interpretation is in line with recent views of the evolution of learning and social learning strategies, through the fine tuning of their attentional and learning parameters (Goldstein et al. 2010; Lotem and Halpern 2012; Heyes 2012; Leadbeater 2015; Lotem et al. 2017; Prat et al. 2022).

Finally, our results suggest that in contrast to novel actions, which are difficult to learn without social demonstration, food-related cues can be learned quite easily through both social and asocial learning. Thus, we may expect that when it comes to food-related cues, sparrows will be free to rely more heavily on either individual or social information depending on strategic considerations. Indeed, individual experience is assumed to capture expected value more reliably than occasional observations (Ilan et al. 2013), which may explain why sparrows rely more heavily on private information when social and private information are in conflict (Ilan et al. 2013; Truskanov and Lotem 2015; Aljadeff et al. 2020). On the other hand, and as mentioned earlier, when the learning task was made more difficult by adding operant learning to cue learning (Marković et al. 2023), sparrows relied more heavily on social information, which is consistent with a suggested social learning strategy known as “copy when uncertain” (Laland 2004; Smolla et al. 2016).

## Supplementary Information

Below is the link to the electronic supplementary material.

Supplementary file1 (PDF 860 KB)

## Data Availability

The data that support the findings of this study are available at ‘Figshare’ data repository: https://figshare.com/ndownloader/files/45432130. DOI: 10.6084/m9.figshare.25532419.
