# Supplementary material for: Social demonstration of colour preference improves the learning of associated demonstrated actions
Source: Anim Cogn. 2024 Apr 9;27(1):31. doi: 10.1007/s10071-024-01865-7 (PMC11004050; doi:10.1007/s10071-024-01865-7)
Supplement: Supplementary file 1 — Supplementary file1 (PDF 860 KB) [file 10071_2024_1865_MOESM1_ESM.pdf]

Electronic supplementary materials (for Zurek et al., Social demonstration of colour preference improves the learning of associated demonstrated actions)

Fig. S1

Figure 2 of the main text divided into 2020 (N=29) and 2021 (N=13) data sets, showing similar results. Since we did not run the No demonstration group in 2021, only the data of the Action-only and Paired demonstration group is shown for 2021.

2020 2021

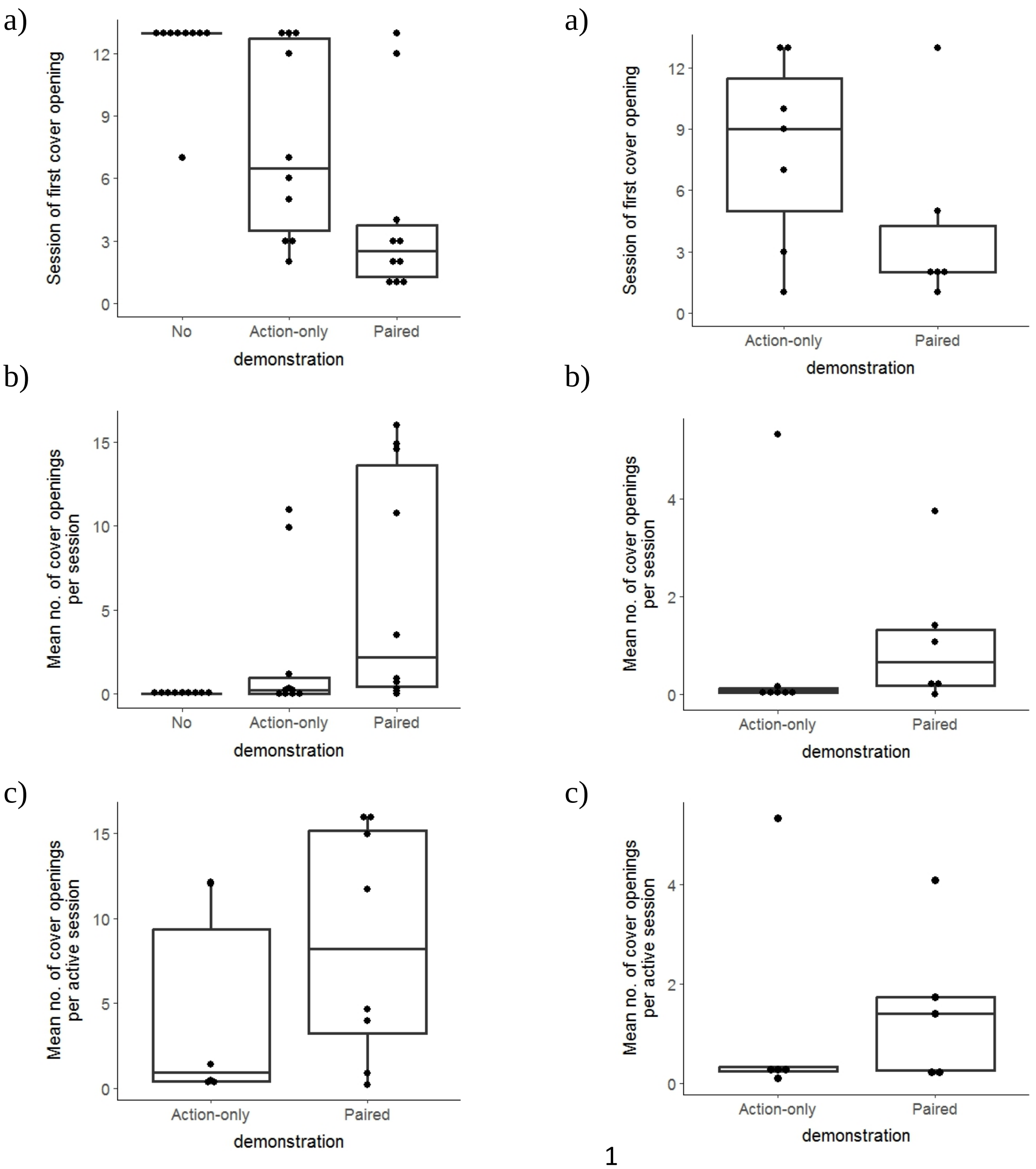

Fig. S2

The method of assigning a proximity score by dividing the observer's cage into four quarters (using the three central bars of the celling of the cage to mark the visual border between them). For example, the proximity score of the observer in this particular video frame would be 3 (see further details in the main text).

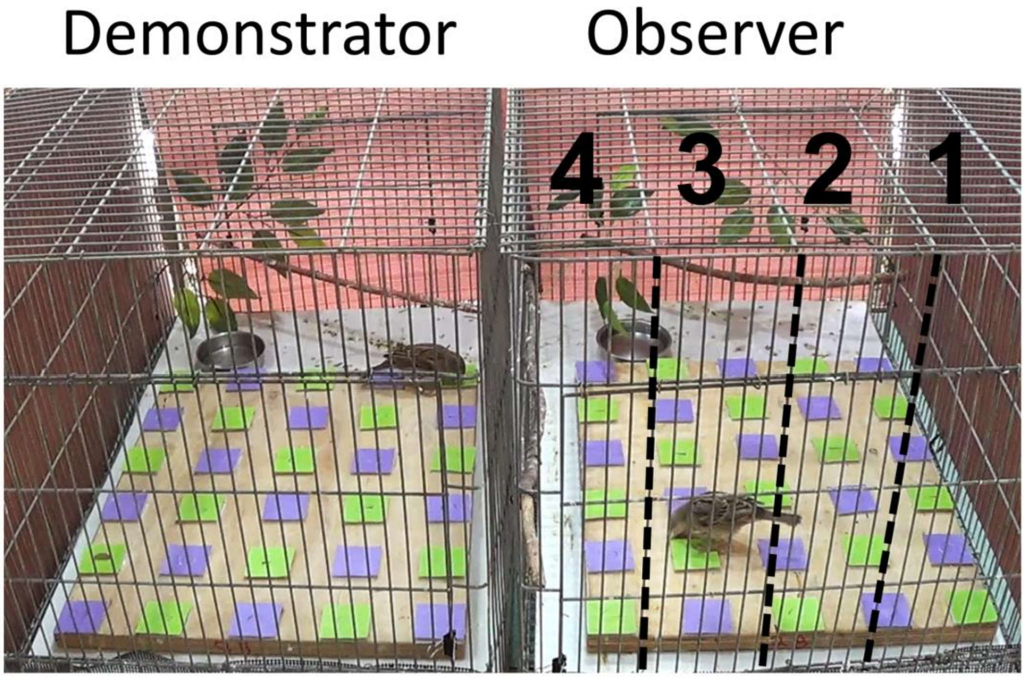

Fig. S3

Individuals' first landing on the foraging grid in the three experimental groups (No demonstration, Action-only demonstration, and Paired demonstration). **a)** The distribution of birds who first landed on the grid during session 1, session 2, or during any of the following sessions (3 to 12). **b)** Latency to first landing on the grid (in seconds) from the beginning of the first session (latencies for birds who first landed on session 2 were calculated by adding the duration of session 1). Boxplots show median, mean (as ×), and 25th and 75th percentiles; the whiskers indicate the values within 1.5 times the interquartile range, and dots are the outliers.

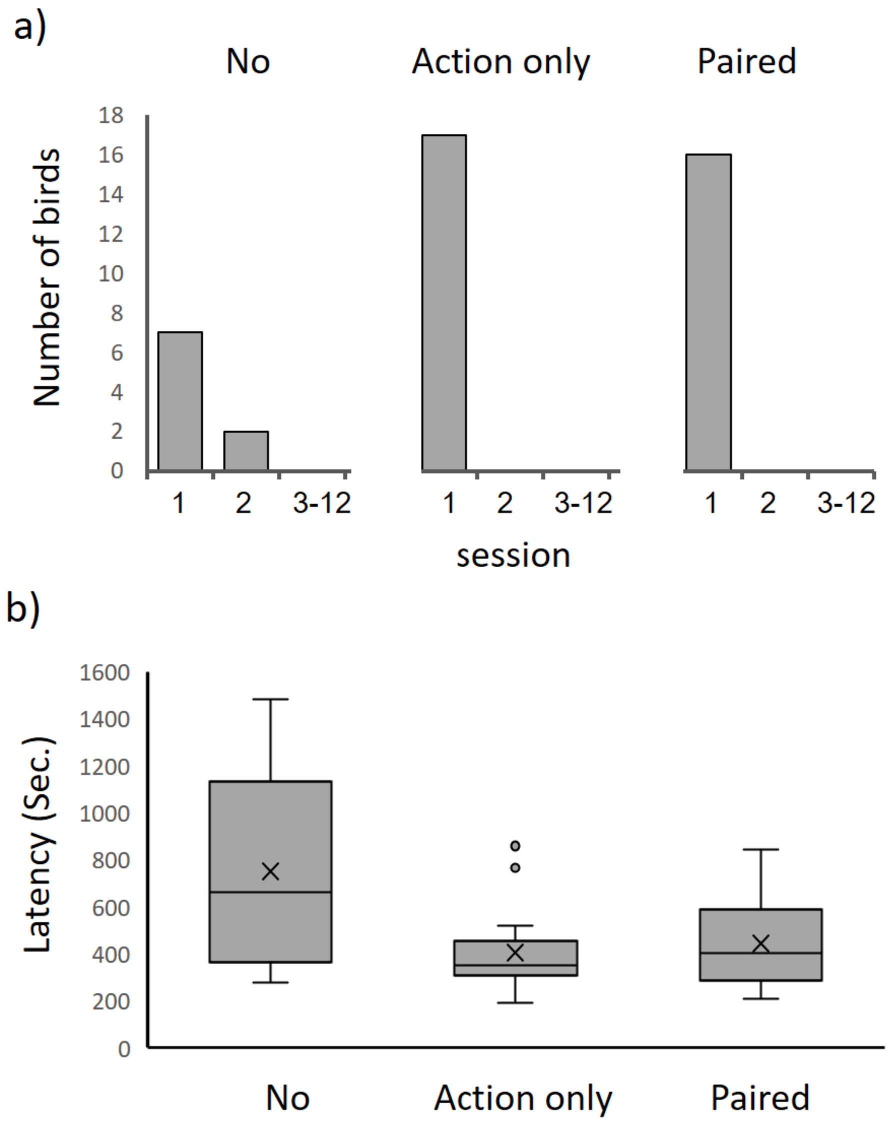

Table S1.

Statistical GLM models from Table 1 of the main text, in which the interaction was not significant (Models C, D, and E), after removing the interaction term and showing similar results. The models test the effect of experimental group and number of demonstrations on different measures of social learning success (model C includes all three groups, models D and E include only the Paired and Action-only groups)*

Model	*N*	Response variable	Fixed effects	*df*	$\chi 2$	*P*
C	41	Mean number of pecks per session	Experimental group	2	8.6346	**0.013**
			Number of demonstrations	1	5.1259	**0.024**
D	32	Mean number of pecks per session	Experimental group	1	3.982	**0.046**
			Number of demonstrations	1	4.026	**0.045**
E	24	Mean number of pecks per active sessions	Experimental group	1	2.337	0.126
			Number of demonstrations	1	4.664	0.052

* We used Quasi-Poisson distribution for these models.

Table S2

The results of the statistical GLM models of Table 1 (of the main text) after including the "Latency to first landing on the grid" as an additional predictor in each of them. The effect of Latency is never significant but the main results do not change even when it is included. The models test the effect of experimental group, number of demonstrations, and Latency, on three different measures of social learning success (models A and C includes all three groups, models B, D, and E includes only the Paired and Action-only groups)*

Model	*N*	Response variable	Fixed effects	*df*	$\chi2$	*P*
A	41	Session of first opening	Experimental group	2	40.228	< **0.001**
			Number of demonstrations	1	0.104	0.746
			Latency	1	0.005	0.944
			Interaction	2	8.639	**0.013**
B	32	Session of first opening	Experimental group	1	17.865	< **0.001**
			Number of demonstrations	1	0.024	0.878
			Latency	1	2.592	0.107
			Interaction	1	6.054	**0.014**
C	41	Mean number of pecks per session	Experimental group	2	10.806	**0.005**
			Number of demonstrations	1	4.876	**0.027**
			Latency	1	1.652	0.199
			Interaction	2	1.831	0.400
D	32	Mean number of pecks per session	Experimental group	1	6.113	**0.013**
			Number of demonstrations	1	3.941	**0.047**
			Latency	1	1.390	0.238
			Interaction	1	1.472	0.225
E	24	Mean number of pecks per active sessions	Experimental group	1	2.640	0.104
			Number of demonstrations	1	4.664	0.063
			Latency	1	1.102	0.294
			Interaction	1	1.101	0.294

* We used Poisson distribution for models A and B, and Quasi-Poisson distribution for models C, D and E.

Table S3

The results of GLM models B, D, and E of Table 1 (of the main text) after including the Year (2020 vs. 2021) as a categorical factor (upper panel), and after including both Year and the interaction between Year and Experimental Group (lower panel). The effects of Year and Year and Group interaction were not significant and the main results do not change when they are included in the model (compare with Table 1 of the main text). All models compare the Paired and the Action-only groups for which there is data from both 2020 and 2021. We used Poisson distribution for model B, and Quasi-Poisson distribution for models D and E.

Model	*N*	Response variable	Fixed effects	*df*	$\chi 2$	*P*
B	32	Session of first opening	Experimental group	1	16.468	< **0.001**
			Number of demonstrations	1	0.113	0.737
			Year	1	2.841	0.594
			Interaction (G*D)	1	9.070	**0.003**
D	32	Mean number of pecks per session	Experimental group	1	4.711	**0.029**
			Number of demonstrations	1	0.032	0.857
			Year	1	0.081	0.776
			Interaction (G*D)	1	2.595	0.107
E	24	Mean number of pecks per active sessions	Experimental group	1	3.994	**0. 046**
			Number of demonstrations	1	2.678	0.102
			Year	1	0.197	0.657
			Interaction (G*D)	1	0.207	0.649
B	32	Session of first opening	Experimental group	1	16.468	< **0.001**
			Number of demonstrations	1	0.094	0.759
			Year	1	2.841	0.594
			Interaction (G*D)	1	9.055	**0.003**
			Interaction (G*Y)	1	0.028	0.868
D	32	Mean number of pecks per session	Experimental group	1	4.520	**0.033**
			Number of demonstrations	1	0.026	0.873
			Year	1	0.078	0.780
			Interaction (G*D)	1	2.485	0.115
			Interaction (G*Y)	1	0.008	0.931
E	24	Mean number of pecks per active sessions	Experimental group	1	4.237	**0.040**
			Number of demonstrations	1	3.465	0.062
			Year	1	0.209	0.647
			Interaction (G*D)	1	0.103	0.748
			Interaction (G*Y)	1	1.542	0.214

(G*D) - Interaction of Group and number of Demonstrations; (G*Y) – Interaction of Group and Year.

Table S4

Discrimination learning success: colour preference data for observers that opened at least 5 covers in the Action-only and Paired demonstration groups

Individual id	Group	Rewarding color	Number opened	Number of correct choices*	Proportion correct*	Binomial test
94562	Action-only	Green	96	91	0.9479167	p<0.0001
15364	Action-only	Green	113	103	0.9115044	p<0.0001
15371	Action-only	Purple	14	11	0.7857143	p=0.0307
15471	Action-only	Green	61	46	0.754098	p<0.0001
10661	Paired	Purple	9	9	1	p=0.0038
15276	Paired	Green	115	104	0.9043478	p<0.0001
15349	Paired	Green	168	153	0.9107143	p<0.0001
15379	Paired	Purple	42	37	0.8809524	p<0.0001
15369	Paired	Purple	147	134	0.9115646	p<0.0001
15380	Paired	Green	174	167	0.9597701	p<0.0001
15510	Paired	Green	17	14	0.823529	p=0.0127
15559	Paired	Purple	14	12	0.857143	p=0.0129
15568	Paired	Green	45	45	1	p<0.0001

* Only the first 15 intact wells visited in a session (or less, if less than 15 intact wells were visited) were used for this analysis because later choices may be biased by the lack of available intact wells of the preferred colour (see Methods).

Table S5

Opening attempts made before first successful cover opening by the learners (observers) of the Paired demonstration group.

Individual id	Total attempts before first opening	Correct attempts	Proportion correct	Correct >0.5
94565	3	0	0	
10661	10	8	0.8	+
15276	23	17	0.7391304	+
15354	12	7	0.5833333	+
15362	27	14	0.5185185	+
15351	2	1	0.5	=
15349	4	4	1	+
15379	1	1	1	+
15369	4	2	0.5	=
15380	2	2	1	+
15521	19	14	0.736842	+
15515	46	34	0.73913	+
15473	19	9	0.473684	
15559	66	55	0.833333	+
15568	42	16	0.380952	
